# Multiple cutaneous melanomas associated with gastric and brain metastases*

**DOI:** 10.1590/abd1806-4841.20164374

**Published:** 2016

**Authors:** Lara Caroline Grander, Fernanda Cabral, Alice Paixão Lisboa, Gabrielle Vale, Carlos Baptista Barcaui, Juan Manuel Pineiro Maceira

**Affiliations:** 1 Universidade do Estado do Rio de Janeiro (UERJ) – Rio de Janeiro (RJ), Brazil; 2 Private clinic – São Paulo (SP) - Brazil; 3 Private clinic – Rio de Janeiro (RJ), Brazil; 4 Universidade Federal do Rio de Janeiro (UFRJ) – Rio de Janeiro (RJ), Brazil

**Keywords:** Cyclin-dependent kinase inhibitor protein, Cyclin-dependent Kinases, Melanoma, Neoplasm metastasis

## Abstract

The occurrence of multiple primary melanomas in a single individual is rare. Most commonly, malignant melanocytic lesions subsequent to the initial diagnosis of melanoma are secondary cutaneous metastases. We report a patient with gastrointestinal bleeding from gastric metastasis of cutaneous melanoma. During clinical evaluation and staging, we discovered a brain metastasis associated with 3 synchronous primary cutaneous melanomas. We suggest the research on the mutation in the cyclin-dependent kinase inhibitor 2A (CDKN2A) (INK4a) in such cases. We also emphasize the importance of clinical examination and dermoscopy of the entire tegument, even after a malignant melanocytic lesion is identified.

## INTRODUCTION

Multiple primary melanomas are rarely seen, with an incidence estimated at 1.7-4%.^1,2^ The first article reporting the presence of more than one synchronous primary melanoma dates back to 1952. The occurrence was observed in 16 patients, with an incidence of 1.3%.^3^ In most cases, only two primary melanomas were observed.^3^ The present case shows the unique simultaneous diagnosis of three primary cutaneous melanomas with the evidence of concomitant gastric and brain metastases.

## CASE REPORT

**Figure 1:**
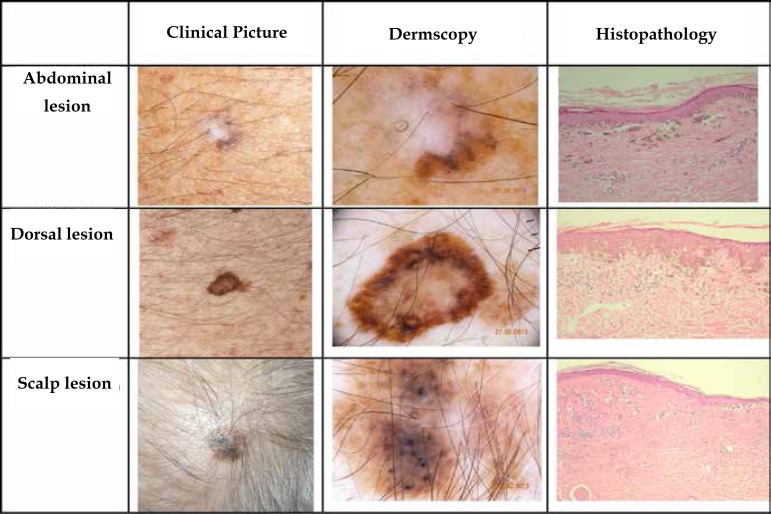
Clinical, dermoscopic, and histopathological correlation of the lesions. **Abdominal lesion:** hypochromic macula with hyperpigmentation spots; dermoscopy – irregular pigment network, multiple blue spots and scarring white areas; histopathology – asymmetric and hyperpigmented melanocytic proliferation predominantly intraepidermal associated with inflammatory changes suggesting spontaneous regression phenomenon. **Back lesion:** polychromic macula; dermoscopy – global multicomponent pattern; histopathology – partial occupation of the epidermis by hyperplastic and hyperpigmented melanocytic cells. **Scalp lesion:** polychromatic macula with ill-defined borders; dermoscopy – multicomponent global pattern; Histopathology – anaplastic melanocytic cell proliferation in the dermal-epidermal junction and in the upper dermis.

We report a 67-year-old Caucasian patient who was diagnosed with melanoma after a gastric biopsy by endoscopy that revealed upper gastrointestinal bleeding. He reported no family history of melanoma or neoplasms. Total gastrectomy was performed. Histopathological study of the sample revealed gastric melanoma with transmural impairment of the gastric wall and angiolymphatic invasion. CT scans of the abdomen, pelvis, and chest during staging showed no abnormalities. However, skull imaging suggested the presence of brain metastases. After the diagnostic confirmation by histopathology, the patient underwent radiosurgery. We requested the opinion of a dermatologist in order to identify the primary site of the metastases. Physical and dermoscopic examination revealed three suspicious melanocytic lesions (Figure 1). One of them was located on the right hypochondrium, featuring a hyperchromic macula with a hypochromic side area. Dermoscopy of the lesion revealed an irregular pigment network, multiple bluish spots, and scarring white areas. On the back, we observed a hyperchromic macula with well-defined edges and polychromy. Dermoscopy revealed a multicomponent global pattern composed of an irregular pigment network, stretch marks, irregular blackish amorphous areas, and granularity. The third lesion, on the scalp, was a hyperchromic and polychromatic macula with ill-defined borders. Dermoscopy of the lesion revealed irregular pigment network, irregular globules, spots, and amorphous areas, gray blue veil, and scarring white areas, forming a multicomponent global pattern. The patient had noticed none of the lesions by then and reported no history of cutaneous lesion excision. Lesion were surgically removed and histopathological exam was compatible with superficial spreading melanoma. The lesion on the back was a melanoma *in situ*. The melanoma located in the abdominal region had inflammatory changes suggestive of partial regression, Breslow 0.10 mm, and Clark level II. The lesion on the scalp was Breslow 0.60 mm and Clark level III (Table 1). The patient died four months after the diagnosis.

**Table 1:** Histopathological features of melanomas

	Abdomen	Back	Scalp
Histological type	Superficial spreading	Superficial spreading	Superficial spreading
Breslow	0.10 mm	Non-applicable	0.60 mm
Ulceration	Absent	Absent	Absent
Mitotic rate	0/mm^2^	Non-applicable	0/mm^2^
Clark level	II	I	III

## DISCUSSION:

Patients who develop melanoma are at higher risk of presenting new primary melanomas.^4^ The cumulative probability is estimated at 0.99% in the first year, 2.06% in the following five years, and 5.34% over the next twenty years.^4^ Incidence rates are higher for men, elderly, and patients with a family history of melanoma.^4,5^

Primary melanoma of the gastrointestinal tract is extremely rare and is suggested in the absence of other primary cutaneous melanomas or metastases. Diagnosis of a metastatic melanocytic tumor of the stomach in a living patient is also unusual. Clinical picture includes anemia, abdominal pain, weight loss, apparent or occult gastrointestinal bleeding, and abdominal mass.^6^ Approximately 60% of patients who die of melanoma have gastrointestinal metastases identified by autopsy.^6^ The most common sites in descending order are: small intestine, colon, and stomach, the latter is affected by 5-7% of cases.^6^ Moreover, brain metastases are common in individuals with melanoma.^7^ Among the solid tumors, melanoma is a major risk factor for the development of brain metastases which occur in about 40% of the patients with advanced stages of the disease.^7,8^ Considering that the melanomas in our patient were less than 1mm thick, we believe that the spontaneous regression phenomenon would be responsible for the gastric metastases. Although unlikely, there is no claim that the gastric lesion is a primary melanoma, but the histopathological features and the presence of another metastatic site suggest metastasis.

About 5-10% of melanomas are hereditary.^9,10^ Patients with three or more primary invasive melanomas and/or families with at least one invasive melanoma and two or more other diagnoses of invasive melanoma and/or pancreatic cancer among first or second degree relatives on the same side of the family should be candidates for genetic consultation.^9^ Mutations in the gene cyclin-dependent kinase inhibitor 2A (CDKN2A) (INK4a) have been found in 40% of the hereditary melanomas, making its analysis essential in the suggested cases.^9,10^ It is noteworthy that the incidence of positive association between the CDKN2A mutation and the development of melanoma varies according to country/region and the studied families.^9^ It is postulated that this variation may be attributed to environmental factors, the coexistence of other genetic variations, or type of mutation.^9^ Other genes, such as cyclin-dependent kinase 4 (CDK4) and cyclin-dependent kinase inhibitor 2A/p14 (CDKN2A/ARF) may be studied in patients who have a strong family history and are negative for CDKN2A mutation.^9^ Although our patient had not been screened for the mutation and had no family history of melanoma or pancreatic cancer, considering genetic tests in his offspring is a valid option, as they fall into the category of families diagnosed with one case of invasive melanoma and two other cases of melanoma.

We aim to report a rare case of three primary melanomas synchronous with gastric and brain metastases diagnosed during life and to underscore the importance of a complete skin physical examination and detailed dermoscopy in patients with melanoma metastases.

We suggest that, even after identifying a suspected malignant skin lesion, scrutiny be extended to the entire body surface because, as reported in our case, more than one melanoma can coexist and should be promptly identified.
